# Normal Light-Dark and Short-Light Cycles Regulate Intestinal Inflammation, Circulating Short-chain Fatty Acids and Gut Microbiota in *Period2* Gene Knockout Mice

**DOI:** 10.3389/fimmu.2022.848248

**Published:** 2022-03-18

**Authors:** Yongkang Zhen, Ling Ge, Qiaoyun Xu, Liangyu Hu, Wenjun Wei, Jiantao Huang, Juan J. Loor, Qingyong Yang, Mengzhi Wang, Ping Zhou

**Affiliations:** ^1^College of Animal Science and Technology, Yangzhou University, Yangzhou, China; ^2^State Key Laboratory of Sheep Genetic Improvement and Healthy Production, Xinjiang Academy of Agricultural Reclamation Sciences, Shihezi, China; ^3^Human and Animal Physiology, Wageningen University & Research, Wageningen, Netherlands; ^4^Mammalian Nutrition Physiology Genomics, Department of Animal Sciences and Division of Nutritional Sciences, University of Illinois, Urbana, IL, United States

**Keywords:** *Per2* knockout, light–dark cycle, short-chain fatty acids, circadian rhythm, inflammation, 16S rRNA sequencing

## Abstract

Regular environmental light–dark (LD) cycle-regulated period circadian clock 2 (*Per2*) gene expression is essential for circadian oscillation, nutrient metabolism, and intestinal microbiota balance. Herein, we combined environmental LD cycles with *Per2* gene knockout to investigate how LD cycles mediate *Per2* expression to regulate colonic and cecal inflammatory and barrier functions, microbiome, and short-chain fatty acids (SCFAs) in the circulation. Mice were divided into knockout (KO) and wild type (CON) under normal light–dark cycle (NLD) and short-light (SL) cycle for 2 weeks after 4 weeks of adaptation. The concentrations of SCFAs in the serum and large intestine, the colonic and cecal epithelial circadian rhythm, SCFAs transporter, inflammatory and barrier-related genes, and Illumina 16S rRNA sequencing were measured after euthanasia during 10:00–12:00. KO decreased the feeding frequency at 0:00–2:00 but increased at 12:00–14:00 both under NLD and SL. KO upregulated the expression of *Per1* and *Rev-erbα* in the colon and cecum, while it downregulated *Clock* and *Bmal1*. In terms of inflammatory and barrier functions, KO increased the expression of *Tnf-α*, *Tlr2*, and *Nf-κb p65* in the colon and cecum, while it decreased *Claudin* and *Occludin-1*. KO decreased the concentrations of total SCFAs and acetate in the colon and cecum, but it increased butyrate, while it had no impact on SCFAs in the serum. KO increased the SCFAs transporter because of the upregulation of *Nhe1*, *Nhe3*, and *Mct4*. Sequencing data revealed that KO improved bacteria *α*-diversity and increased Lachnospiraceae and Ruminococcaceae abundance, while it downregulated *Erysipelatoclostridium*, *Prevotellaceae UCG_001*, *Olsenella*, and *Christensenellaceae R-7* under NLD in KO mice. Most of the differential bacterial genus were enriched in amino acid and carbohydrate metabolism pathways. Overall, *Per2* knockout altered circadian oscillation in the large intestine, KO improved intestinal microbiota diversity, the increase in Clostridiales abundance led to the reduction in SCFAs in the circulation, concentrations of total SCFAs and acetate decreased, while butyrate increased and SCFAs transport was enhanced. These alterations may potentially lead to inflammation of the large intestine. Short-light treatment had minor impact on intestinal microbiome and metabolism.

## Introduction

Rhythmic alterations in physiology, metabolism, behavior, and circulation of nutrients with a 24-h cycle are ubiquitous in mammals. The circadian clock is formed by the adaption of environmental light–dark (LD) cycles (1). As the core circadian clock gene, period circadian clock 2 (*Per2*) gene is wildly distributed in the central nervous system such as the brain and spinal cord, or surrounding organs, such as the skin and gastrointestinal tract (2). Central Per2 protein regulates the rhythmic expression of Per2 in the limbic system, which is a key factor whereby the suprachiasmatic nucleus (SCN) coordinates the operation of the peripheral circadian clock (3). Mutations in *Per2* will shorten the circadian rhythm or even lead to disappearance of circadian oscillation, and inhibition of the circadian clock producer transcriptional-translational feedback loops (TTFLs) axis (4–6).

A recent report revealed that a mutation in the *PER2* in reindeer caused a loss of binding ability with cryptochrome 1 (CRY1), resulting in arrhythmicity, thus adapting the reindeer to the drastic LD changes in polar regions (7). *Per2* is closely related to body fat metabolism and physiological functions of the liver (8). Studies have shown that the deletion of *Per2* in mice can cause glucocorticoid imbalance. The diurnal rhythm of neuroendocrine peptide αMSH is disrupted in *Per2*-deficient mice, which leads to circadian eating disorders such as night-eating syndrome, which gradually leads to obesity (9). *Per2* knockout mice showed more severe liver fibrosis, cholestasis, or infarction under toxic conditions than wild-type mice (10). In addition, the LD cycle is an important factor that leads to the circadian rhythm oscillation. Long-time light reduces the amplitude of SCN oscillation of mice at night and enhances the feeding behavior during the day (11). In addition, sleep and wake cycle abnormalities of shift workers increase food intake at night and reduce light exposure, which in turn triggers metabolic disorders due to inadaptability to circadian rhythms, leading to a significant increase in the incidence of obesity, diabetes, and coronary heart disease (12). It is reported that the intrinsically photosensitive retinal ganglion cells (ipRGCs) can sensitively respond to the changes in the external light cycle, which adjusts the SCN to receive the signal input from the retina to synchronize the circadian rhythm with the LD cycles (13, 14).

Approximately 4,000 strains of bacteria colonize the intestine of mammals, which constitute the dominant intestinal microbiota and play an important role in regulating digestion, absorption, immunity, and growth (15). Moreover, studies have found that the number and colonization of intestinal microbiota also have circadian rhythms (16, 17). In 17% of cecum, bacterial relative abundance oscillates with the rhythm, and in 15% in feces (18, 19), the absolute abundance of Firmicutes phylum fluctuates slightly with the rhythm and the absolute abundance of Bacteroidetes phylum fluctuates greatly during the day (20). Rhythm disorders can cause intestinal microbiota imbalance, which is characterized by alterations in the composition and function of bacteria, affecting the disappearance of circadian oscillations and health. For example, fecal microbiota transplantation from rhythmically disturbed mice into germ-free mice can lead to obesity and glucose metabolism disorders (18). High-fat diet induces imbalance of the intestinal microbiota and the disappearance of the abundance of Lachnospiraceae oscillation in mice, which leads to the disturbance of the synthesis and metabolism of short-chain fatty acids (SCFAs) (21). Several studies also demonstrated that the gut microbiome can regulate the expression of *Per2* by varieties of metabolites; for examples, *Clostridium sporogenes* is reported to be involved in modulating the circadian oscillation *of Per2* in the peripheral tissues by producing 3-(4-hydroxyphenyl)propionic acid (4-OH-PPA) and 3-phenylpropionic acid (PPA) (22). Gut microbiota-derived SCFAs and lactate can also modulate the phase of *Per2* (23), and the circadian pattern of *Per2* expression in the hepatic organoid altered upon treatment with butyrate from gut microbiota (21). Research shows that deacetylase 3 (Hdac3) is a key protein that integrates microbiota and circadian rhythms. It can mediate the interaction of bacteria and the circadian clock to cause the circadian oscillation of histone acetylation and deacetylation, thereby affecting feeding and metabolism-related gene expression (24). In addition, intestinal epithelial toll-like receptors (Tlrs) and myeloid differentiation primary response gene 88 (Myd88) protein can also identify specific microbial groups and affect the rhythmic expression of nuclear receptor *Rev-erbα*, thus regulating the circadian transcription factor *Nfil3* expression (25).

LD cycles are the main factors that impact biological rhythms (26). Irregular LD cycles can trigger intestinal metabolic disorders and induce obesity, diabetes, and non-alcoholic fatty liver diseases (10). Except for the extreme LD cycles of constant darkness (0 h of light and 24 h of darkness) or constant light (24 h of light and 0 h of darkness), the LD cycle of 8L:16D is used most frequently as the environmental LD cycle model, which has also been studied to simulate the trans-equatorial jet lag as short-light (SL) cycle (27). SL can also induce circadian rhythm disorder; for example, the rhythm of the brain and muscle ARNT-like 1 (*Bmal1*) gene is lost when the rhythm phase is advanced by 6 h in mice (28); the increased diseases such as metabolic homeostasis imbalance (obesity, diabetes, and cardiovascular), decreased immunity, and increased incidence of tumors and depression of long-term jet lag among shift workers may also result from SL cycle (29, 30). Ikegami et al. (31) found a strong period circadian clock 1 (*Per1*) expression late at night and strong *Cry1* expression at midnight in wild-type mice under SL. In addition, there was a high *Cry1* expression in *Per2* mutant mice at midnight under SL, which suggested that the LD cycle of 8L:16D also causes rhythm disorders. Besides, SL cycle is more related to the reproduction of animals (32), and food restricted under SL showed significant regression of the reproductive system of California mice (33).

Herein, we hypothesized that *Per2* can respond to different environmental LD cycles (NLD and SL), thus regulating circadian oscillation and impacting the gut microbiome and nutrient metabolism. Therefore, *Per2* knockout (KO) and wild-type (CON) mice were managed under NLD and SL, respectively, for 2 weeks after adaption for 4 weeks, in order to investigate how *Per2* regulates colonic and cecal inflammatory functions, barrier functions, gut microbiome, and SCFAs circulation in response to different LD cycles.

## Materials and Methods

### Ethics Statement

All animal experiments were performed according to the ethical policies and procedures approved by the Animal Care and Use Committee of Yangzhou University, Jiangsu, China (Approval no. SYXK (Su) 2017-0044).

### Mouse Management and Experimental Design

The heterozygous C57BL/6N mice with systemic *Per2* gene knockout (*Period2^+/−^*) based on CRISPR/Case9 technology were provided by Biocytogen Biotechnology Co., Ltd. (Beijing, China). Mice were then sent to the Institute of Neuroscience, Chinese Academy of Science, non-human primate research platform (Suzhou, China), to expand the scale of reproduction, in order to obtain a sufficient number of positive homozygous mice (*Period2^−/−^*, KO) and wild-type mice (*Period2^+/+^*, CON). Each 8-week-old mouse was in good health, similar in body size and initial body weight. KO and CON mice were then randomly divided into alternating photoperiods of normal light–dark cycle treatment (NLD, 12 h of light and 12 h of darkness) and short-light cycle treatment (SL, 8 h of light, 16 h of darkness) (31, 33, 34); each treatment contained six mice. Mice were managed in a single cage in an environmentally controlled warehouse that allowed for manipulating LD cycles according to regulations; the LD cycles were controlled with an LED light strip of 150–200 lx and temperature of 4,500–5,000 K. The adaptation period of LD cycle and diet for mice was 4 weeks. Feeding of each mouse was recorded from 0:00–2:00, 6:00–8:00, 12:00–14:00, and 18:00–20:00 during the trial period using surveillance cameras. Composition of the commercial maintenance pellet feed consisted of corn, soybean, wheat, chicken meal, fish meal, and vegetable oil. Total energy content was 3,616 kcal/kg with a protein content of 18.6% (20.6% of calorie %), carbohydrate of 61% (67.4% of calorie %), and fat of 4.8% (12.0% of calories %). The trough, drinking fountain, and litter were changed weekly; adequate drinking water and pellet feed were provided. The experiment lasted for 14 days after allowing the mice to adapt to the diet and light conditions for 4 weeks.

### Sample Collection

Mice were fasted 1 day before sampling; then, blood was collected from the retroorbital sinus for measurements of concentrations of SCFAs. Mice were then anesthetized with ether and euthanized by spinal dislocation during 10:00–12:00 a.m. The colon and cecum tissues were harvested, rinsed in phosphate buffer solution (PBS), and rapidly frozen in liquid nitrogen for measurements of the expressions of circadian rhythm, SCFAs transporter, and inflammatory- and barrier-related genes following a previously described protocol (8). Colonic and cecal contents were stripped along the outer wall of the intestine and stored in liquid nitrogen for concentration of SCFAs and microbiome analysis.

### Total RNA Extraction

Total RNA was extracted from colonic and cecal epithelial tissues using the FastPure Cell/Tissue Total RNA Isolation Kit V2 (RC112, Vazyme, Nanjing, China). Concentration and purity of total RNA were determined with a NanoDrop spectrophotometer (Thermo Fisher Scientific, Waltham, MA, USA). Reverse transcription referred to FastKing gDNA Dispelling RT Super Mix (Tiangen, Beijing, China). Reverse transcription reaction system was as follows: 5× FastKing-RT Super Mix, 4 µl; total RNA, 1,000 ng; and RNase-free ddH_2_O to make the volume of 20 µl. Reaction procedure was set as 42°C for 15 min and 95°C for 3 min following the manufacturer’s instructions.

### Real-Time PCR

The reverse transcription gDNA samples were used as templates for real-time PCR (RT-PCR) using the 2× TSINGKE Master qPCR Mix (SYBR Green I) (TSE201, Tsingke, Beijing, China) in an ABI7500 (Thermo Fisher) sequence detector. The reaction system for PCR was as follows: qPCR Mix, 10 µl; forward primer, 0.8 µl (10 µM); reverse primer, 0.8 µl (10 µM); 50× ROX Reference Dye, 0.4 µl; and ddH_2_O to make the volume of 20 µl. PCR reaction procedure was set as 95°C for 60 s, 40 cycles of 95°C for 10 s and 60°C for 30 s, and, ultimately, the test was set as 95°C for 15 s, 60°C for 60 s, 95°C for 30 s, and 60°C for 15 s. The standard curve method and QuantStudio™ 7 Flex Real-Time PCR Software (Applied Biosystems, Foster, CA, USA) were used for data analysis. Relative expression of the target gene was calculated using 2^−ΔΔCt^ methods (35). Specific primers used for RT-PCR are shown in **Supplementary Table S1**.

### Protein Extraction

Total protein was extracted from colonic and cecal epithelial tissues using radioimmunoprecipitation assay (RIPA) lysis buffer (R0010, Solarbio, Beijing, China), which contained 1 mM dilution of phenylmethylsulfonyl fluoride (78830, Sigma-Aldrich, St. Louis, MO, USA). Total protein was extracted according to the manufacturer’s instructions, and the concentration of protein was determined with the Enhanced BCA Protein Assay Kit (Beyotime Institute of Biotechnology, Nantong, Jiangsu, China). Protein was denatured at 95°C for 10 min prior to Western blotting.

### Western Blot

Western blot method was used to determine the protein expression of Per2. Details of Western blot were reported previously by our laboratory (36). Briefly, primary antibodies used in this work were Per2 (GTX129688, GeneTex, Irvine, CA, USA), and Gapdh (GTX100118, GeneTex, Irvine, CA, USA). After incubating with primary antibody, the membranes were incubated with the horseradish peroxidase (HRP)-conjugated anti-rabbit IgG secondary antibody (GTX213110-0, GeneTex, Irvine, CA, USA). The polyvinylidene fluoride (PVDF) membranes were developed using SuperSignal West Femto Substrate Trial Kit (No. 34094, Thermo Fisher Scientific, Waltham, MA, USA). The visualization of PVDF membranes was performed by FulorChem HD2 (ProteinSimple, San Francisco, CA). The band intensities were measured using Image J software (v1.8.0). The target protein abundance was normalized with the intensity of Gapdh.

### Determination of SCFAs

For colonic and cecal contents, 0.5 g of mixtures of each treatment was used, 1.25 ml of ultrapure water per sample was mixed with the contents, and they were vortexed for 5 min, then centrifuged at 4°C, 13,400×*g* for 10 min. The supernatant was collected and filtered through a 0.25-μM syringe filter. A total of 0.4 ml metaphosphoric acid (contained 20% of 60 mmol/L crotonic acid as internal standard) was added, and then, the entire contents were vortexed, centrifuged, and filtered, and the supernatant was collected for subsequent analyses. A standard mix (0.4 μl) contains 55.06 mmol/L of acetate, 15.84 mmol/L of propionate, 4.25 mmol/L of isobutyrate, 8.57 mmol/L of butyrate, 3.58 mmol/L of isovalerate, and 3.56 mmol/L of valerate, and test samples were mixed and ran through a CP-WAX capillary column (length, 30 m; inner diameter, 0.53 mm; and film thickness, 1 μm) in a gas chromatograph (GC-9A, Shimadzu, Kyoto, Japan). The SCFAs were determined according to the preliminary method of our laboratory (37). Briefly, 0.4 μl of standard product was collected, and the solution with a CP-WAX capillary column was tested (length, 30 m; inner diameter, 0.53 mm; and film thickness, 1 μm) by using the gas chromatography. Program settings and calculations of concentrations were according to a previous protocol (38). The relative correction factor of SCFAs was calculated by the peak area of the standard and the internal standard, and the concentration was calculated based on the peak area.

Blood was kept at room temperature for 30 min and centrifuged at 1,000×*g* for 15 min. The serum was collected for concentrations of SCFAs measurements. Then, 200 µl of serum; a 200-µl mixture of organic solvents composed of N-butanol, tetrahydrofuran, and acetonitrile in a 50:30:20 ratio; 40 µl of HCl 0.1M; 20 mg of citric acid; and 40 mg of sodium chloride were mixed. The microtubes were shook vigorously using the vortex stirrer for 1 min and centrifuged at 13,400×*g* at room temperature for 10 min. The supernatant was transferred, and concentrations of SCFAs were measured according to a previous protocol (39).

### DNA Extraction and Illumina Sequencing

In each treatment, a 0.5-g mixture of colonic and cecal contents was taken, and the TIANamp Stool DNA Kit (DP328, Tiangen) was used to extract total microbial DNA. The concentration and purity of total DNA were determined by the NanoDrop spectrophotometer (Thermo Fisher Scientific, Waltham, MA, USA). Illumina Hiseq2500-PE250 sequencing platform was used to determine the abundance and diversity of the intestinal content microbiota. The method amplified the bacterial 16S ribosomal RNA (rRNA) region using the following primers: primer (319F: 5′-ACTCCTACGGGAGGCAGCAG-3′; 806R: 5′-GGACTACHVGGGTWTCTAAT-3′) target V3–V4 hypervariable region was used for sequencing (40). Sequencing was conducted at Genepioneer Biotechnologies Co., Ltd., Nanjing, China. The 16S rRNA gene amplicon sequencing data generated during the current study were submitted to National Center for Biotechnology Information (NCBI) under BioProject PRJNA750583.

### 16S rRNA Sequencing Data Processing

Paired-end reads generated from Illumina platforms were processed and merged using FLASH software (v1.2.7) (41). Then, high-quality clean sequence tags were obtained by removing lower quality and shorter lengths. Usearch software (Uparse v6.0.307) was used to cluster all the reads of each sample into operational taxonomic units (OTUs) with a sequence similarity level of 97% (42). Representative sequences of each OTUs were screened for further annotation. Matched bacterial 16S rRNA classification information was obtained from SILVA (Release115) database (43). Relative abundances of representative bacteria were calculated at the phylum, class, order, family, and genus levels. QIIME software (V1.7.0) was used to calculate the Alpha diversity index (Chao1, ACE, and Shannon and Simpson Index) (44). Beta diversity was calculated using principal coordinate analysis (PCoA) based on Unweight Unifrac distance. Linear discriminant analysis effect size (LEfSe) was used to compare the marker species under the genus classification (LDA score >3.5) (45). Finally, the PICRUSt2 (v1.1.0) database was used to predict the function of the microbial community based on high-quality sequences and annotated *via* Kyoto Encyclopedia of Genes and Genomes (KEGG) database (46).

### Statistical Analysis

Relative expression of genes and protein, concentrations of SCFAs, and bacterial α-diversity based on 16S rRNA sequencing were subjected to two-way ANOVA analysis using SPSS 13.0 (SPSS, Inc., Chicago, IL, USA) software. GraphPad Prism 6.0 software was used to draw histograms. Results are presented as means ± standard error of the mean (SEM) (* denotes *p* < 0.05, significant difference; ** or ***denotes *p* < 0.01 or *p* < 0.001, extremely significant difference). Bacterial taxa analyses were plotted using the R package “ggplot2” based on the modified OTUs data. The Venn diagrams were analyzed using the R package “VennDiagram.” PCoA diagram was carried out by R package “vegan.” The bubble diagram of pathway enrichment analysis was made using the R package “ggplot2.” The bacterial cluster results were carried out by R package “pheatmap.” The R version was 4.0.2, and R packages were described previously (47, 48).

## Results

### Daily Feeding Rhythm of Mice

**Figure 1:**
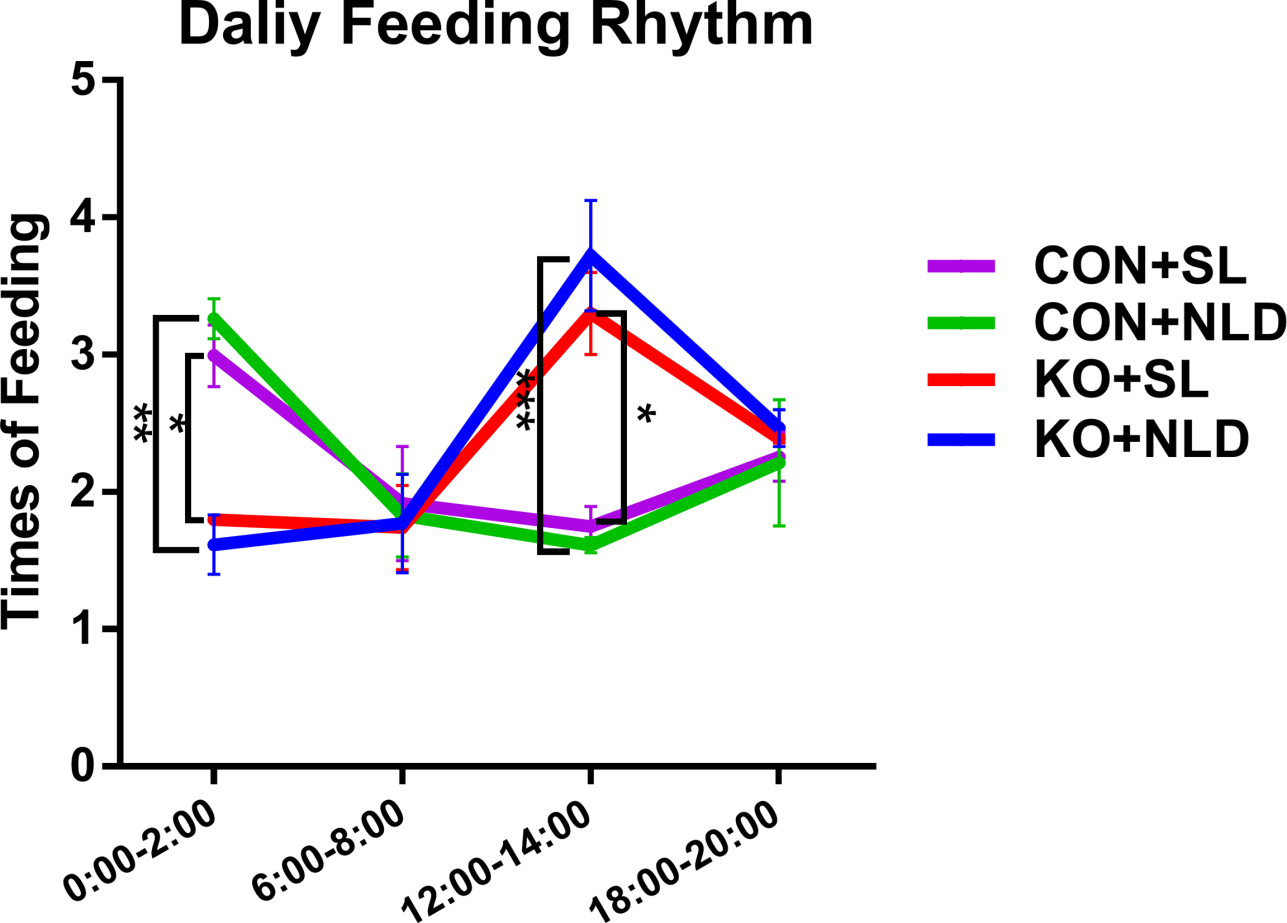
Daily feeding rhythm of KO and CON mice under NLD and SL. KO, *Per2* gene knockout (*Period2*^−/−^) mice; CON, wild-type (*Period2*^+/+^) mice; NLD, normal light–dark cycle treatment of 12 h of light and 12 h of darkness; SL, short-light treatment of 8 h of light and 16 h of darkness. Recorded the times of feeding of each mouse from 0:00–2:00, 6:00–8:00,12:00:14:00, and 18:00–20:00 during the trial period using surveillance cameras. Statistical analyses were conducted using two-way ANOVA. *p < 0.05, significant difference; ** or ***p < 0.01 or *p* < 0.001, extremely significant difference. Results are presented as means ± SEM (n = 4 per group).

Data indicated that the feeding frequency of *Per2* knockout mice was significantly decreased compared with CON mice at 0:00–2:00 (*p* < 0.05 or *p* < 0.01) but increased at 12:00–14:00 (*p* < 0.05 or *p* < 0.001) both under NLD and SL (**Figure 1**). KO did not impact the feeding frequency at 6:00–8:00 and 18:00–20:00 (*p* > 0.05). Short-light treatment also did not alter the daily feeding rhythm compared with NLD of KO and CON mice (*p* > 0.05). The feeding rhythm of CON mice showed trends that were higher at night but lower during the day, while KO mice showed the opposite.

### Concentrations of SCFAs in Serum

Data indicated that *Per2* knockout did not have a significant impact on the concentrations of total SCFAs, acetate, propionate, butyrate, and acetate/propionate (*p* > 0.05) compared with CON mice (**Table 1**). Short-light treatment also did not impact all SCFAs indicators compared with CON mice (*p* > 0.05). There was no interaction effect on the serum SCFAs indicators (*p* > 0.05).

**Table 1:** Concentrations of SCFAs in serum.

Serum SCFAs	LD Cycles	KO	CON	*p*-value		
				*p*_KO_	*p*_LD_	*p*_INT_
Total SCFAs, mmol/L	NLD	2.20 ± 1.11	1.68 ± 0.51	0.339	0.177	0.987
	SL	1.45 ± 0.06	0.95 ± 0.35			
Acetate, mmol/L	NLD	1.72 ± 0.71	1.52 ± 0.48	0.446	0.173	0.768
	SL	1.25 ± 0.10	0.82 ± 0.29			
Propionate, mmol/L	NLD	0.31 ± 0.26	0.13 ± 0.05	0.226	0.434	0.560
	SL	0.17 ± 0.05	0.11 ± 0.04			
Butyrate, mmol/L	NLD	0.17 ± 0.14	0.03 ± 0.01	0.311	0.287	0.188
	SL	0.02 ± 0.01	0.04 ± 0.02			
Acetate/Propionate	NLD	4.87 ± 0.65	12.06 ± 2.21	0.135	0.961	0.104
	SL	8.74 ± 2.84	8.39 ± 1.17			

KO, Per2 gene knockout (Period2^−/−^) mice; CON, wild-type (Period2^+/+^) mice; NLD, normal light–dark cycle treatment of 12 h of light and 12 h of darkness; SL, short-light treatment of 8 h of light and 16 h of darkness. Statistical analyses were conducted using two-way ANOVA. p<0.05, significant difference; p<0.01 or p<0.001, extremely significant difference; p>0.05, without a difference. INT, the interaction between two factors. Results are presented as means ± SEM (n = 2–4 per group).

### Concentrations of SCFAs in Colonic and Cecal Contents

Compared with CON mice, *Per2* knockout significantly reduced the concentrations of total SCFAs (*p* < 0.001), acetate (*p* = 0.001), propionate (*p* = 0.011), and increased butyrate (*p* = 0.021), but did not impact isobutyrate, isovalerate, valerate, and acetate/propionate (*p* > 0.05) in colonic contents (**Table 2**). Short-light treatment did not impact the concentrations of SCFAs (*p* > 0.05), except reducing isobutyrate (*p* < 0.001) and isovalerate (*p* = 0.031) compared with NLD. The interaction between two treatments did not impact the concentration of SCFAs (*p* > 0.05). In terms of the concentration of SCFAs in cecal content, KO reduced total SCFAs (*p* = 0.020) and acetate (*p* < 0.01) but increased butyrate (*p* = 0.001); no differences were observed in propionate, isobutyrate, isovalerate, valerate, and acetate/propionate (*p* > 0.05). Short light did not impact SCFAs indicators (*p* > 0.05) compared with NLD in cecal content. Besides, the interaction between two treatments significantly impacted the concentration of propionate (*p* < 0.01).

**Table 2:** Concentrations of SCFAs in colonic and cecal contents.

Intestinal SCFAs	LD cycles	KO	CON	*p*-value		
				*p*_KO_	*p*_LD_	*p*_INT_
Colonic content						
Total SCFAs, mmol/L	NLD	7.40 ± 0.35	14.62 ± 1.32	<0.001	0.607	0.362
	SL	6.72 ± 1.27	15.17 ± 1.48			
Acetate, mmol/L	NLD	4.87 ± 0.36	10.34 ± 1.28	0.001	0.709	0.550
	SL	4.57 ± 1.51	11.63 ± 1.41			
Propionate, mmol/L	NLD	1.13 ± 0.11	3.22 ± 0.45	0.011	0.892	0.761
	SL	1.23 ± 0.54	2.96 ± 0.69			
Isobutyrate, mmol/L	NLD	0.41 ± 0.06	0.44 ± 0.02	0.489	<0.001	0.913
	SL	0.13 ± 0.01	0.17 ± 0.01			
Butyrate, mmol/L	NLD	0.75 ± 0.14	0.37 ± 0.05	0.021	0.314	0.930
	SL	0.64 ± 0.13	0.24 ± 0.05			
Isovalerate, mmol/L	NLD	0.20 ± 0.01	0.16 ± 0.01	0.446	0.031	0.244
	SL	0.11 ± 0.04	0.12 ± 0.01			
Valerate, mmol/L	NLD	0.04 ± 0.01	0.09 ± 0.02	0.130	0.263	0.439
	SL	0.04 ± 0.01	0.05 ± 0.02			
Acetate/Propionate	NLD	4.44 ± 0.16	3.68 ± 0.23	0.175	0.332	0.330
	SL	3.81 ± 0.30	3.68 ± 0.47			
Cecal content						
Total SCFAs, mmol/L	NLD	20.76 ± 0.23	24.37 ± 0.48	0.020	0.757	0.861
	SL	21.46 ± 0.41	23.33 ± 3.62			
Acetate, mmol/L	NLD	13.86 ± 0.37	17.65 ± 0.59	<0.01	0.620	0.641
	SL	13.90 ± 0.63	18.95 ± 2.14			
Propionate, mmol/L	NLD	3.77 ± 0.17	4.66 ± 0.19	0.163	0.147	<0.001
	SL	4.48 ± 0.11	3.08 ± 0.33			
Isobutyrate, mmol/L	NLD	0.43 ± 0.04	0.45 ± 0.02	0.779	0.908	0.932
	SL	0.40 ± 0.11	0.41 ± 0.08			
Butyrate, mmol/L	NLD	2.19 ± 0.26	1.07 ± 0.13	0.001	0.197	0.749
	SL	2.21 ± 0.28	0.50 ± 0.08			
Isovalerate, mmol/L	NLD	0.26 ± 0.02	0.24 ± 0.02	0.161	0.058	0.399
	SL	0.24 ± 0.01	0.18 ± 0.01			
Valerate, mmol/L	NLD	0.25 ± 0.02	0.30 ± 0.01	0.590	0.649	0.220
	SL	0.23 ± 0.05	0.21 ± 0.05			
Acetate/Propionate	NLD	3.91 ± 0.29	4.06 ± 0.25	0.606	0.984	0.665
	SL	3.22 ± 0.30	4.43 ± 1.33			

KO, Per2 gene knockout (Period2^−/−^) mice; CON, wild-type (Period2^+/+^) mice; NLD, normal light–dark cycle treatment of 12 h of light and 12 h of darkness; SL, short-light treatment of 8 h of light and 16 h of darkness. Statistical analyses were conducted using two-way ANOVA. p < 0.05, significant difference; p < 0.01 or p < 0.001, extremely significant difference; p > 0.05, without a difference. INT, the interaction between two factors. Results are presented as means ± SEM (n = 4–6 per group).

### Relative Expression of Colonic and Cecal Epithelial Circadian Rhythm Genes and Per2 Protein

**Figure 2:**
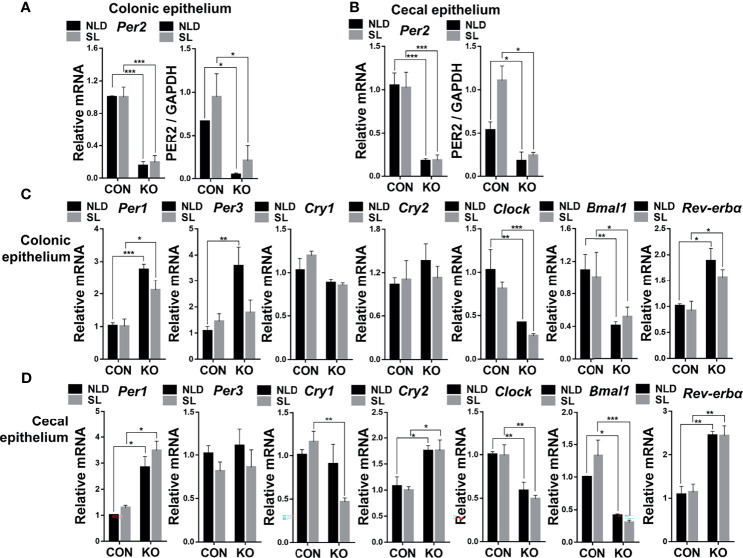
Relative expression of colonic and cecal epithelial circadian rhythm genes and Per2 protein in KO and CON mice under NLD and SL. KO, *Per2* gene knockout (*Period2*^-/-^) mice; CON, wild-type (*Period2*^+/+^) mice; NLD, normal light–dark cycle treatment of 12 h of light and 12 h of darkness; SL, short-light treatment of 8 h of light and 16 h of darkness. Representative charts of the expression levels of Per2 gene and protein of colonic epithelium **(A)** and cecal epithelium **(B)**, *Per1* gene, *Per3* gene, *Cry1* gene, *Cry2* gene, *Clock* gene, *Bmal1* gene, and *Rev-erbα* gene of colonic epithelium **(C)** and cecal epithelium **(D)**. Expressions of circadian genes and Per2 protein were determined in *Per2* knockout and wild-type mice under NLD and SL by PCR and Western blot method. Statistical analyses were conducted using two-way ANOVA. *p < 0.05, significant difference; ** or ***p < 0.01 or *p* < 0.001, extremely significant difference. Results are presented as means ± SEM (n = 6 per group).

Compared with CON mice, mRNA and protein expression of Per2 significantly decreased in *Per2* knockout mice both under NLD and SL in colonic epithelium (**Figure 2A**, **Supplementary Figure S1**) and cecal epithelium (**Figure 2B**, **Supplementary Figure S1**) (*p* < 0.05 or *p* < 0.001). There was a high knockout efficiency of *Per2* gene in colon and cecum tissues. In terms of expression of other circadian rhythm genes during 10:00 to 12:00 a.m., KO upregulated expression of *Per1* and *Rev-erbα* gene but downregulated circadian locomotor output cycles kaput (*Clock*) and *Bmal1* both under NLD and SL. The KO also increased the expression of period circadian clock 3 (*Per3*) gene under NLD in colonic epithelium (**Figure 2C**) (*p* < 0.05, *p* < 0.01, or *p* < 0.001) compared with CON mice. For cecal epithelium, KO upregulated expression of *Per1*, cryptochrome 2 (*Cry2*), and *Rev-erbα* but downregulated expression of *Clock* and *Bmal1* genes both under NLD and SL. The KO also downregulated expression of *Cry1* gene under SL (**Figure 2D**) (*p* < 0.05, *p* < 0.01, or *p* < 0.001) compared with CON mice. Short-light treatment did not impact expression of colonic and cecal epithelial circadian rhythm genes (*p* > 0.05) compared with NLD.

### Relative Expression of Colonic and Cecal Epithelial SCFAs Transporter Genes

**Figure 3:**
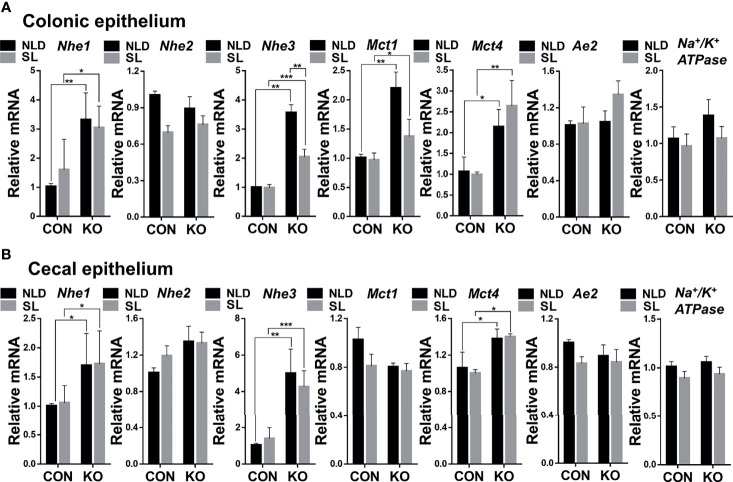
Relative expression of colonic and cecal epithelial SCFAs transporter genes in KO and CON mice under NLD and SL. KO, *Per2* gene knockout (*Period2*^−/−^) mice; CON, wild-type (*Period2*^+/+^) mice; NLD, normal light–dark cycle treatment of 12 h of light and 12 h of darkness; SL, short-light treatment of 8 h of light and 16 h of darkness. Representative charts of the expression levels of *Nhe1* gene, *Nhe2* gene, *Nhe3* gene, *Mct1* gene, *Mct4* gene, *Ae2* gene, and *Na^+^/K^+^ ATPase* gene of colonic epithelium **(A)** and cecal epithelium **(B)**. Expressions of SCFAs transporter genes were determined in *Per2* knockout and wild-type mice under NLD and SL by PCR method. Statistical analyses were conducted using two-way ANOVA. *p < 0.05, significant difference; ** or *** *p* < 0.01 or *p* < 0.001, extremely significant difference. Results are presented as means ± SEM (n = 6 per group).

Compared with CON mice, *Per2* knockout significantly upregulated expression of Na^+^/H^+^ exchanger 1 (*Nhe1*), Na^+^/H^+^ exchanger 3 (*Nhe3*), monocarboxylic acid transporters 1 (*Mct1*), and monocarboxylic acid transporters 4 (*Mct4*) (*p* < 0.05, *p* < 0.01, or *p* < 0.001) but did not impact expression of Na^+^/H^+^ exchanger 2 (*Nhe2*), anion exchanger *2* (*Ae2*), and *Na^+^/K^+^ ATPase* (*p* > 0.05) both under NLD and SL in colonic epithelium (**Figure 3A**). Short-light treatment further down-regulated expression of colonic epithelial *Nhe3* gene compared with NLD in KO mice (*p* < 0.01) (**Figure 3A**). For cecal epithelium, KO upregulated expression of *Nhe1*, *Nhe3*, and *Mct4* genes (*p* < 0.05, *p* < 0.01, or *p* < 0.001) but did not impact expression of *Nhe2*, *Mct1*, *Ae2*, and *Na^+^/K^+^ ATPase* (*p* > 0.05) both under NLD and SL in cecal epithelium (**Figure 3B**). Short light did not alter expression of SCFAs transporter genes in cecal epithelium compared with NLD (**Figure 3B**) (*p* > 0.05).

### Relative Expression of Colonic and Cecal Epithelial Inflammatory and Barrier Genes

**Figure 4:**
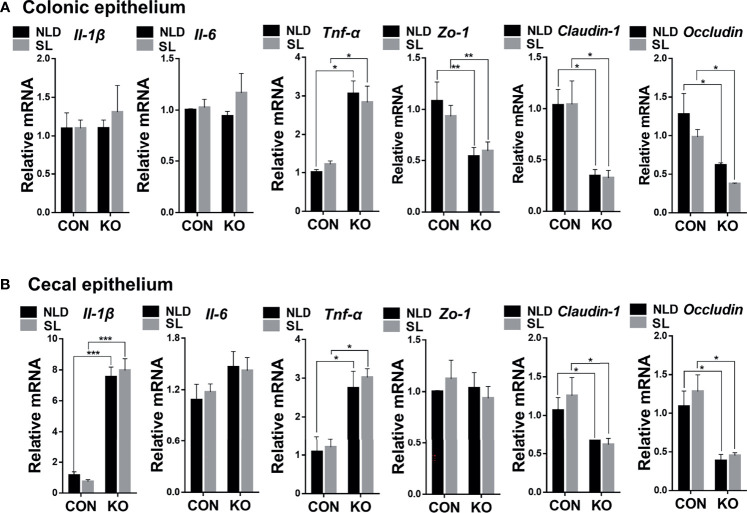
Relative expression of colonic and cecal epithelial inflammatory and barrier genes in KO and CON mice under NLD and SL. KO, *Per2* gene knockout (*Period2*^−/−^) mice; CON, wild-type (*Period2*^+/+^) mice; NLD, normal light–dark cycle treatment of 12 h of light and 12 h of darkness; SL, short-light treatment of 8 h light and 16 h darkness. Representative charts of the expression levels of *Il-1β* gene, *Il-6* gene, *Tnf-α* gene, *Zo-1* gene, *Claudin-1* gene, and *Occludin* gene of colonic epithelium **(A)** and cecal epithelium **(B)**. Expressions of inflammatory and barrier genes were determined in *Per2* knockout and wild-type mice under NLD and SL by PCR method. Statistical analyses were conducted using two-way ANOVA. *p < 0.05, significant difference; ** or *** *p* < 0.01, or *p* < 0.001, extremely significant difference. Results are presented as means ± SEM (n = 6 per group).

Compared with CON mice, *Per2* knockout significantly upregulated expression of tumor necrosis factor *α* (*Tnf-α*) gene, while it downregulated expression of tight junction protein 1 (*Zo-1*), *Claudin-1*, and *Occludin* genes (*p* < 0.05 or *p* < 0.01) but did not impact the expression of interleukin 1*β* (*Il-1β*) and interleukin 6 (*Il-6*) genes (*p* > 0.05) both under NLD and SL in colonic epithelium (**Figure 4A**). In terms of cecal epithelial inflammatory and barrier genes, KO significantly upregulated expression of *Il-1β* and *Tnf-α*, while it downregulated expressions of *Claudin-1* and *Occludin* (*p* < 0.05 or *p* < 0.001). However, it did not impact expression of *Il-6* and *Zo-1* genes (*p* > 0.05) both under NLD and SL (**Figure 4B**). Short light did not alter expression of colonic and cecal epithelial inflammatory and barrier genes in colonic and cecal epithelium compared with NLD (*p* > 0.05).

### Relative Expression of Colonic and Cecal Epithelial Inflammatory Pathway-Related Genes

**Figure 5:**
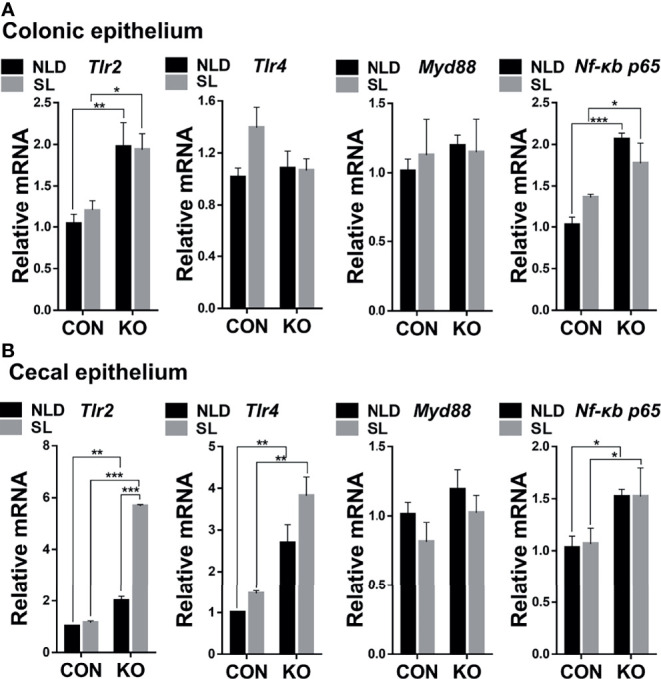
Relative expression of colonic and cecal epithelial inflammatory pathways-related genes in KO and CON mice under NLD and SL. KO, *Per2* gene knockout (*Period2*^−/−^) mice; CON, wild-type (*Period2*^+/+^) mice; NLD, normal light–dark cycle treatment of 12 h of light and 12 of h darkness; SL, short-light treatment of 8 h light and 16 h darkness. Representative charts of the expression levels of *Tlr2* gene, *Tlr4* gene, *Myd88* gene, and *Nf-κb p65* gene of colonic epithelium **(A)** and cecal epithelium **(B)**. Expressions of inflammatory pathways-related were determined in *Per2* knockout and wild-type mice under NLD and SL by PCR method. Statistical analyses were conducted using two-way ANOVA. *p < 0.05, significant difference; ** or *** *p* < 0.01 or *p* < 0.001, extremely significant difference. Results are presented as means ± SEM (n = 6 per group).

Compared with CON mice, *Per2* knockout significantly upregulated expression of toll-like receptor 2 (*Tlr2*) and nuclear factor *κ*b (*Nf-κb*) *p65* genes (*p* < 0.05, *p* < 0.01, or *p*<0.001) but did not impact expression of toll-like receptor 4 (*Tlr4*) and *Myd88* genes (*P* > 0.05) both under NLD and SL in colonic epithelium (**Figure 5A**). In terms of cecal epithelial pathway-related genes, KO significantly upregulated expression of *Tlr2*, *Tlr4*, and *Nf-κb p65* genes (*p* < 0.05, *p* < 0.01, or *p* < 0.001) but did not impact expression of *Myd88* (*p* > 0.05) both under NLD and SL (**Figure 5B**). Short light did not alter expression of colonic and cecal epithelial inflammatory pathway-related genes compared with NLD (*p* > 0.05), except upregulating the expression of *Tlr2* gene of KO mice in cecal epithelium (*p* < 0.001).

### Bacterial Sequencing OTUs and Diversity in Intestinal Contents

**Figure 6:**
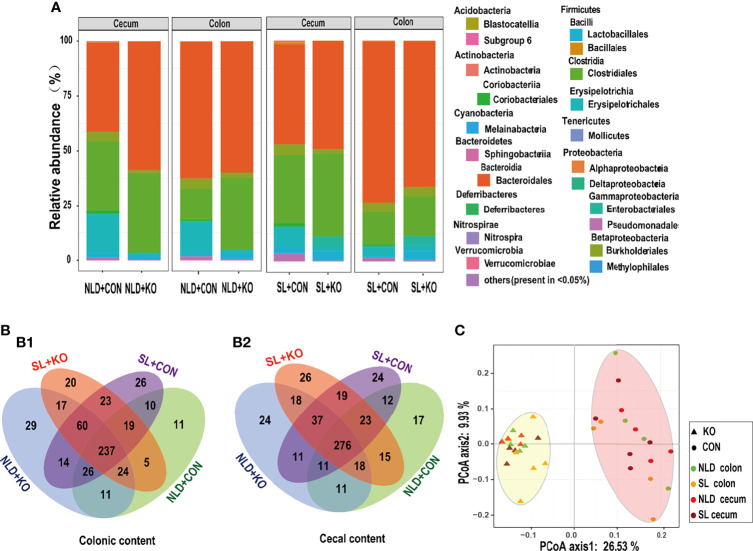
Bacterial diversities and compositions in KO and CON mice under NLD and SL. KO, *Per2* gene knockout (*Period2*^−/−^) mice; CON, wild-type (*Period2*^+/+^) mice; NLD, normal light–dark cycle treatment of 12 h of light and 12 h of darkness; SL, short-light treatment of 8 h of light and 16 h of darkness. **(A)** Compositions of colonic and cecal microbiota at the phylum, family, and order levels in KO and CON mice under NLD and SL, the bacterial relative abundance in <0.05% were belonged to others. **(B)** Venn diagrams based on OTUs in colonic content (B1) and cecal content (B2), the number of unique OTUs were represented by the unoverlapped portion. **(C)** Principal coordinate analysis plots of unweighted UniFrac distances of microbiota in colonic and cecal contents in KO and CON mice under NLD and SL. n = 6 per group for 16S rRNA sequencing.

**Table 3** and **Figure 6** underscore the alterations in the diversity of intestinal microbiota of KO and CON mice under NLD and SL. The 16S rRNA sequencing coverage exceeded 99% of each group (**Table 3**). The Venn diagram revealed that numbers of unique bacterial OTUs of colonic contents for NLD+KO, NLD+CON, SL+KO, and SL+CON were 29, 11, 20, and 26, respectively, and the shared OTUs numbers was 237 (**Figure 6B**1). Besides, the unique bacterial OTUs of cecal contents for the above groups were 24, 17, 26, and 24, respectively, and the shared OTUs number was 276 (**Figure 6B**2). Meanwhile, **Figure 6A** indicates that different treatments had different relative abundance at the level of bacterial phyla and class (partial orders) classification. The bacteria with higher abundance mainly included Bacteroidales order of Bacteroidetes phylum, Clostridiales and Erysipelotrichales order of Firmicutes phylum, etc. β-Diversity calculated by PCoA diagram based on Unweight Unifrac distance (**Figure 6C**) revealed that the bacteria of KO mice were completely separated from CON mice on the first axis, and the NLD and the SL were not significantly separated, which indicated that *Per2* knockout significantly altered intestinal bacterial β-diversity, while short-light treatment did not have an impact. Lastly, the *α*-diversity (**Table 3**) showed that the Chao 1, ACE, Shannon, and Simpson indices of KO mice were significantly higher than those of CON mice (*p*<0.05) both under NLD and SL of colonic and cecal microbiome, except that KO had no impact on the Simpson index of cecal content (*p* > 0.05). Short light did not impact bacterial α-diversity (*p* > 0.05).

**Table 3:** Sequencing coverage and α-diversity indicators of gut microbiome.

α-diversity	LD cycles	KO	CON	*p*-value		
				*p*_KO_	*p*_LD_	*p*_INT_
Colonic content						
Coverage, %	NLD	99.89 ± 0.01	99.88 ± 0.01	0.912	0.740	0.328
	SL	99.88 ± 0.01	99.90 ± 0.01			
Chao 1	NLD	459.43 ± 3.92	370.93 ± 14.64	0.003	0.250	0.738
	SL	437.40 ± 13.85	331.69 ± 10.43			
ACE	NLD	458.33 ± 7.75	330.68 ± 15.39	0.002	0.683	0.226
	SL	423.86 ± 15.46	348.52 ± 14.09			
Shannon	NLD	5.86 ± 0.28	5.04 ± 0.03	0.007	0.589	0.857
	SL	5.77 ± 0.22	4.85 ± 0.38			
Simpson	NLD	0.96 ± 0.01	0.90 ± 0.01	0.006	0.220	0.428
	SL	0.95 ± 0.01	0.86 ± 0.04			
Cecal content						
Coverage, %	NLD	99.89 ± 0.01	99.88 ± 0.01	0.109	0.683	0.878
	SL	99.89 ± 0.02	99.87 ± 0.01			
Chao 1	NLD	450.15 ± 13.68	395.16 ± 5.08	<0.001	0.720	0.326
	SL	466.46 ± 15.19	387.41 ± 3.58			
ACE	NLD	458.04 ± 7.58	387.71 ± 4.85	<0.001	0.393	0.707
	SL	454.87 ± 8.11	379.76 ± 2.10			
Shannon	NLD	5.79 ± 0.09	5.87 ± 0.03	0.039	0.433	0.006
	SL	5.98 ± 0.11	5.58 ± 0.02			
Simpson	NLD	0.95 ± 0.01	0.95 ± 0.02	0.997	0.516	0.750
	SL	0.96 ± 0.01	0.95 ± 0.01			

KO, Per2 gene knockout (Period2^−/−^) mice; CON, wild-type (Period2^+/+^) mice; NLD, normal light–dark cycle treatment of 12 h of light and 12 h of darkness; SL, short-light treatment of 8 h of light and 16 h of darkness. ACE, abundance-based coverage estimator. Statistical analyses were conducted using two-way ANOVA. p < 0.05, significant difference; p < 0.01 or p < 0.001, extremely significant difference; p > 0.05, without a difference. INT, the interaction between two factors. Results are presented as means ± SEM (n = 6 per group).

### Taxonomic Differences in Microbial Genus Level in Intestinal Contents

**Figure 7:**
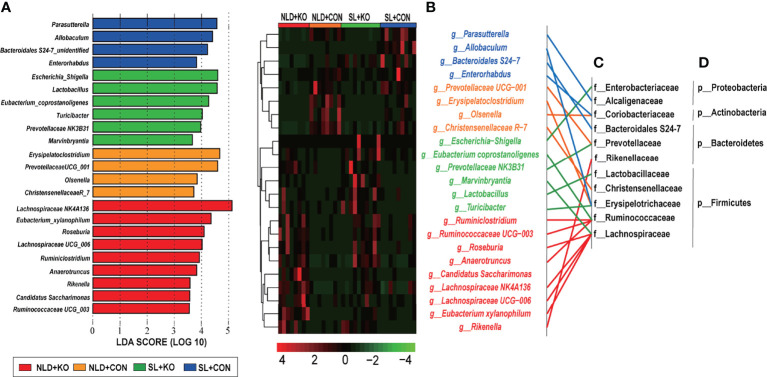
LEfSe analysis and cluster heatmap of in intestinal differential microbial genus classifications in KO and CON mice under NLD and SL. KO, *Per2* gene knockout (*Period2*^−/−^) mice; CON, wild-type (*Period2*^+/+^) mice; NLD, normal light–dark cycle treatment of 12 h of light and 12 h of darkness; SL, short-light treatment of 8 h of light and 16 h darkness. **(A)** Linear discriminant analysis (LDA) plus effect size (LEfSe) at bacterial genus levels in KO and CON mice under NLD and SL, only LDA score in over than 3.5 was marked. **(B)** Cluster heatmap of differential bacterial genus levels in KO and CON mice under NLD and SL. **(C)** Classification the differential bacterial genus in family levels. **(D)** Classification the differential bacterial genus in phylum levels. n = 6 per group for 16S rRNA sequencing.

Significant taxonomic differences in bacterial genus classification among four treatments were analyzed using LEfSe (**Figure 7A**). LEfSe results were visualized using a taxonomy bar chart, and only LDA scores over 3.5 were marked. At the bacterial genus level, *Lachnospiraceae NK4A136*, *Eubacterium_xylanophilum*, *Roseburia*, *Lachnospiraceae UCG_006*, *Ruminiclostridium*, *Anaerotruncus*, *Rikenella*, *Candidatus Saccharimonas*, and *Ruminococcaceae UCG_003* genus were enriched in NLD+KO; *Erysipelatoclostridium*, *Prevotellaceae UCG_001*, *Olsenella*, and *Christensenellaceae R_7* genus were enriched in NLD+CON; *Escherichia Shigella*, *Lactobacillus*, *Eubacterium coprostanoligenes*, *Turicibacter*, *Prevotellaceae NK3B31*, and *Marvinbryantia* genus were enriched in SL+KO; and *Parasutterella*, *Allobaculum*, *Bacteroidales S24-7 unidentified*, and *Enterorhabdus* genus were enriched in SL+CON. The cluster results (**Figure 7B**) revealed that the relative abundance of significant taxonomic differences in bacterial genus of each group was higher than that of other groups. Under NLD+KO, most of the differential bacterial genus belonged to Lachnospiraceae family and Ruminococcaceae family, while the other differential bacterial genus belonged to Erysipelotrichaceae family, Prevoteellaceae, Lactobacillaceae family, etc. (**Figure 7C**), and most of the differential bacterial genus belonged to Bacteroidetes phylum and Firmicutes phylum (**Figure 7D**).

### Prediction of Bacterial Functions *via* PICRUSt2

**Figure 8:**
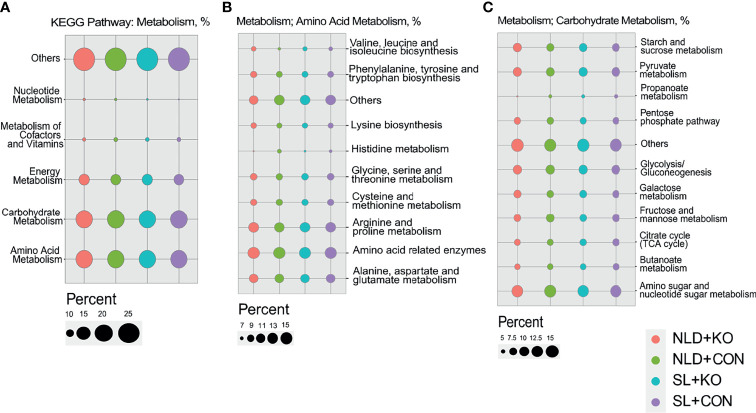
Predicted of bacterial functions of KEGG pathway *via* PICRUSt2 in KO and CON mice under NLD and SL. KO, *Per2* gene knockout (*Period2*^−/−^) mice; CON, wild-type (*Period2*^+/+^) mice; NLD, normal light–dark cycle treatment of 12 h light and 12 h darkness; SL, short-light treatment of 8 h light and 16 h darkness. **(A)** The bacterial functional predictions of KEGG secondary pathway *via* metabolism. **(B)** The amino acid metabolism pathways predicted *via* metabolism. **(C)** The carbohydrate metabolism pathways predicted via metabolism. n = 6 per group for 16S rRNA sequencing.

The bacterial functional predictions of KEGG pathways *via* PICRUSt2 are shown in **Figure 8** and **Supplementary Figure S2**. Prediction of the KEGG primary pathway showed that the identified bacterial genes were potentially related to metabolism, genetic information processing, and environmental information processing (**Supplementary Figure S2**). Based on the metabolism pathway, most of the predicted genes were involved in amino acid and carbohydrate metabolism pathways (**Figure 8A**). The amino acid metabolism pathway was further refined, and we found that functions of the intestinal microbiota were mainly related to pathways such as “alanine, aspartate, and glutamate metabolism” or “arginine and proline metabolism” (**Figure 8B**). The carbohydrate metabolism pathway functions of the intestinal microbiota were mainly related to “amino sugar and nucleotide sugar metabolism,” “pyruvate metabolism,” etc. (**Figure 8C**). Besides, SCFAs metabolism pathways such as “propanoate metabolism” and “butanoate metabolism” were also enriched (**Figure 8C**).

## Discussion

After systemic *Per2* knockout in mice, the mRNA and protein level of Per2 expression in colonic and cecal epithelium was significantly downregulated, which underscored the high efficiency of *Per2* knockout and the reliability of the results of our work. *Per2* knockout altered intestinal circadian oscillation, for example, KO upregulated the expression of *Per1* and *Rev-erbα* but downregulated *Clock* and *Bmal1* in colonic and cecal epithelium during 10:00−12:00. Our results were consistent with previous reports; Zani et al. (49) reported that lack of *Per2* caused altered expressions of the liver by upregulating *Clock*, *Cry1*, and *Bmal1*, while *Rev-erbα*, *Per1*, and *Cry2* were downregulated at ZT14 in *Per2* mutant mice. This indicated that *Per2* is required for normal circadian expression. However, Ikegami et al. (31) argued that *Per2* is not necessary for the photoperiodic response in mice because the expression profile of arylalkylamine *N*-acetyltransferase, a rate-limiting melatonin synthesis enzyme, was unaffected in the pineal gland of *Per2* mutant mice. They also found that the amplitude of *Per1* and *Cry1* expression was greatly attenuated in the SCN. Our data also indicated that short-light treatment did not impact expression of colonic and cecal epithelial circadian rhythm genes compared with NLD because wild-type mice are fast at adjusting their locomotor activity to a long photoperiod (50), and photoperiodic responses remain unaffected in *Per2*-deficient mice (31). In short, *Per2* plays a critical role in the regulation of circadian locomotor activity rhythms, and the simulation of the jet lag by short-light for a short period may not impact circadian rhythms.

Our data indicated that *Per2* knockout also significantly altered the diversity, structure, and function of large intestinal microbiota; for example, PCoA diagram revealed that *Per2* knockout significantly altered intestinal bacterial β-diversity; the α-diversity indicators (Chao 1, ACE, Shannon, and Simpson indexes) of KO mice were significantly higher than those of CON mice both under NLD and SL. However, Thaiss et al. (18) has reported that the Chao 1 index of fecal microorganisms in *Per1/2* genes knockout mice was significantly lower than that of wild-type mice, with the diversity of the population declining. This is because *Per1/2* gene knockout inhibited the TTFLs axis of mice, which causes disappearance of the rhythm of the intestinal microbiota. As such, it inhibits the diversity of intestinal microbiota (6). In our study, intestinal microbiota was dysregulated but did not completely disappear after the single knockout of *Per2*, thus leading to the increase in the *α*-diversity. The increase in bacterial *α*-diversity may result from the abnormal diurnal feeding rhythm of *Per2* knockout mice; the host responds to the increase in feeding frequency and nutrient metabolism disorder caused by *Per2* knockout by increasing the abundance and diversity of intestinal microbiota, thus reducing inflammations in the gut and liver tissues (9, 51).

The *Per2* knockout under normal LD cycle led to an increase in the abundance of the Lachnospiraceae family such as *Lachnospiraceae NK4A136*, *Eubacterium_xylanophilum*, *Roseburia*, *Lachnospiraceae UCG_006*, and Ruminococcaceae family such as *Ruminiclostridium*, *Anaerotruncus*, and *Ruminococcaceae UCG_003*. Thaiss et al. also indicated that the abundance of the above two bacterial families have obvious circadian rhythms (18), i.e., knockout of *Per2* results in disturbance of the intestinal microbiota and increases their abundance. Moreover, Lachnospiraceae family, Ruminococcaceae family, and *Rikenella* genus of Rikenellaceae family are all reported to be closely related to the production of SCFAs in the intestine (52, 53). Furthermore, the abundance of *Erysipelatoclostridium*, *Prevotellaceae UCG-001*, *Olsenella*, and *Christensenellaceae R-7* genus significantly decreased. Takayasu et al. (54) confirmed that Prevotellaceae has a strong circadian rhythm in human saliva, while it disappeared after isolation, which indicated that the circadian rhythm of the intestinal microbiota is closely related to the physiological state of host. Prevotellaceae also plays a role in butyrate production, and *Per2* knockout may cause the abnormal rhythm of Prevotellaceae (55). In addition, *Erysipelatoclostridium* genus of Erysipelotrichaceae family is the main dominant bacteria in the gut of obese mice. Its functions were closely related to obesity, diabetes, and metabolic syndrome diseases (56), and its abundance is also affected by rhythm disorders. Lastly, *Per2* knockout under SL resulted in the increased abundance of *Escherichia-Shigella*, *Lactobacillus*, *Eubacterium coprostanoligenes*, *Turicibacter*, *Prevotellaceae NK3B31*, and *Marvinbryantia*, while the abundance of *Parasutterella*, *Allobaculum*, *Bacteroidales S24-7*, and *Enterorhabdus* decreased. The differentially affected bacterial genus belongs to different families, and their functions and classifications were also inconsistent. In general, the bacterial genus mainly belonged to Erysipelotrichaceae, Lachnospiraceae, Ruminococcaceae, and Prevotellaceae families, which also have a strong rhythm and were closely related to butyrate production.

A stable microbiota rhythm will drive the host’s circadian rhythm transcription, epigenetics, and metabolites to oscillate, and the destruction of homeostasis affects the host’s physiological functions and increases disease risk (57). For example, it has been previously reported that *Clock/Clock* mutant mice demonstrate altered circadian rhythmicity and display reduced cytokine production from macrophages and arrhythmic inflammatory responses, thus leading to intestinal ecology dysregulation (58). The gut microbiota is involved in a variety of digestive and metabolic functions in the hindgut of mammals and exhibits rhythmicity and oscillates in key metabolic mediators of intestine. These mediators also affect the circadian rhythm of the host, thereby maintaining the stability of the metabolic internal environment (21). The prediction of the bacterial KEGG pathway *via* PICRUSt2 in our study revealed that 50% of bacterial genes were annotated into the metabolism pathway, which mainly included amino acid and carbohydrate metabolism, such as “alanine, aspartate, and glutamate metabolism,” “arginine and proline metabolism,” “cysteine and methionine metabolism,” and “glycine, serine, and threonine metabolism” pathways. Carbohydrate metabolism pathways of “amino sugar and nucleotide sugar metabolism,” “pyruvate metabolism,” “fructose and mannose metabolism” ,and “citrate cycle (TCA)”, etc. were also enriched, which indicated that most bacteria were involved in amino acid, carbohydrate, and energy metabolism. Several previous studies showed that *Per2* interacts with key nuclear receptors (*Pparα*, *Pparγ*, and *Rev-erbα*) (59) and plays an important role in various metabolic processes (49). *Per2* mutant mice are characterized by diminished fasting glycemia, absence of rhythmic hepatic glycogen accumulation, increased plasma insulin levels, and weakened gluconeogenesis in addition to a reduction in fat pad mass and plasma lipid levels (49, 60). *Per2*-KO mice lack a glucocorticoid rhythm and diurnal feeding rhythm, which can lead to obesity when fed a high-fat diet (9, 51). This explained why we observed that the feeding rhythms of CON mice showed trends of increase at night but decreases during the day, while that of KO mice were the opposite, and the feeding frequency of *Per2* knockout mice were significantly decreased at 0:00–2:00 but increased at 12:00–14:00. We also found that KO increased the expression of *Tnf-α*, *Tlr2*, and *Nf-κb p65* but decreased *Claudin* and *Occludin-1* in the colon and cecum, which suggested that alterations in daily feeding behavior and gut microbiome and nutrient metabolism of KO mice may lead to destruction of intestinal barrier function, which may be a potential inflammatory response *via* Nf-κb pathway (61, 62).

It is worth noting that butanoate and propanoate metabolism were enriched among carbohydrate metabolism pathway, which proved that *Per2* knockout altered the metabolism of propionate and butyrate. For further verification, the proportion of SCFAs in the colon and cecum were measured. KO hadno impacts on SCFAs in the serum; the probable reason may be that SCFAs were more energy substance for the intestinal epithelial cells rather than transported into the blood, such as butyrate. KO decreased the concentrations of total SCFAs and acetate in the colon and cecum but increased butyrate. In other words, the increased abundance of Lachnospiraceae and Ruminococcaceae in KO was the main reason for the increased concentrations of butyrate. Previous reports also indicated that the above bacteria were closely related to the synthesis of SCFAs. For example, *Lachnospiraceae NK4A136* and *Rosebuira* genus participated in the production and metabolism of butyrate and maintenance of intestinal barrier functions (63, 64). *Eubacterium xylanophilum* was reported to decompose xylan to produce butyrate (65). Besides, *Ruminiclostridium*, *Ruminococcaceae UCG-003*, and *Anaeotruncus* genus of Ruminococcaceae family, and *Rikenella* genus of Rikenellaceae family all belong to the butyrate-producing bacteria (52, 53), while *Lachnospiraceae UCG-006* genus is reported to be an acetate-producing bacteria (66). Our data revealed that *Per2* knockout led to rhythm disturbances and impacted feeding rhythm. Previous reports also demonstrated that *Per2* knockout significantly altered the diurnal feeding rhythm (9, 51) and induced lipid metabolism disorder (8), thus altering intestinal microbiota and increasing the abundance of Lachnospiraceae and Ruminococcaceae, which in turn metabolized and enhanced synthesis of butyrate but decreased total SCFAs and acetate in the intestine.

The transport and absorption of SCFAs in the epithelium is also worthy of attention. SCFAs mainly exist in the form of anions in the intestine, and they must combine with transporters such as MCT1 to be absorbed into the blood through the basal layer of the epithelium (67). KO increased SCFAs transporter because of the upregulated expression of *Nhe1*, *Nhe3*, *Mct1*, and *Mct4*. The above results indicated that *Per2* knockout can promote the absorption of SCFAs by intestinal epithelium. Previous work in our laboratory revealed that silencing *PER2* in goat rumen epithelial cells (GRECs) resulted in the upregulation of *MCT1* abundance, which was consistent with this study. Meanwhile, the abundance of PER2 mRNA and protein increased when 15 mM sodium butyrate was added to stimulate goat GRECs *in vitro*, indicating that absorption of SCFAs regulated by *PER2* may be mediated by butyrate (68). Some unpublished data by our group also revealed that *PER2* and *MCT1/4* gene displayed the opposite daily rhythm of near cosine function, and their daily expression is negatively correlated in GRECs. Overexpression of *PER2* downregulated the expression of *MCT1/4* in GRECs, and perfusion with SCFAs upregulated the *PER2* but downregulated *MCT1/4* in goat *in vivo*. In addition, H^+^ accumulated in greater amounts after the negative ion state of SCFAs was absorbed by the epithelium, which led to the decrease in pH in intestinal epithelial cells and acidification of the intestinal environment. *Nhe1* and *Nhe3* can mediate the transfer of Na^+^ into the cell and transfer of H^+^ outside the cell, thus, lowering the pH and maintaining the stability of the osmotic pressure inside and outside the cell (69, 70). Consistently, expression levels of *Nhe1* and *Nhe3* in colonic and cecal epithelium were significantly increased in KO, which revealed that *Nhe1* and *Nhe3* regulated the homeostasis of the cellular environment caused by the accumulation of H^+^ after SCFAs absorption. Our data demonstrated that *Per2* knockout enhanced SCFAs absorption in the intestine because of the upregulation expression of transporter genes. However, the production and circulation of SCFAs is a highly complex and dynamic process (71). We predicted that the lower SCFAs may be attributed to the greater absorption rather than more SCFAs production because that KO only increased the abundance of butyrate-producing bacteria, such as Lachnospiraceae and Ruminococcaceae, but had no impact on other SCFAs-producing bacteria. The Wood–Ljungdahl pathway is the most efficient for acetate production, and acetate-producing bacteria account for the majority of the Firmicutes phylum (72), and eventually decreased SCFAs concentrations in the intestine. The impacts of *Per2* knockout on intestinal SCFAs production still need to be further investigated because the SCFAs produced by colonic and cecal bacterial microbiota fermentation are based on the diets (63, 70).

In short, our data are consistent with the hypothesis that *Per2* regulates circadian oscillation in the intestine in response to the normal light–dark cycle. The disorder of feeding rhythm leads to upregulating the abundance of Lachnospiraceae and Ruminococcaceae, which further impacting SCFAs metabolism and transport in *Per2* knockout mice. This may potentially lead to inflammation of the colon and cecum. However, *Per2* had little impact on intestinal microbiome and metabolism according to our data in response to short-light cycle. Subsequent experiments needto focus on the alterations in gut microbiome and various metabolic and inflammation processes caused by five different LD cycles (normal LD cycle, consult light, consult darkness, short-light, and long light treatments) after *Per1/2* gene double knockout. Not only that, we need to monitor the dynamic alterations in microbial and metabolic rhythms during a 48 h period.

## Conclusion

In conclusion, based on the present work, *Per2* knockout dysregulated the TTFLs axis and altered the expression of circadian genes such as upregulating *Per1* and *Rev-erbα* and down-regulating *Bmal1* and *Clock* in colon and cecum tissues of mice. Circadian rhythm disorder further induced the loss of feeding rhythm, which in turn led to alterations in gut microbiome. KO increased the *α*-diversity of gut microbiome, and the abundance of Lachnospiraceae and Ruminococcaceae were significantly upregulated, which further led to altering SCFAs metabolism and transport. Data indicated that KO also increased the concentrations of butyrate but decreased total SCFAs and acetate; the transport was enhanced because of the upregulation of *Nhe1*, *Nhe3*, *Mct1*, and *Mct4* genes. This may potentially lead to inflammation of the large intestine. However, short-light treatment had little impact on intestinal microbiome and metabolism according to our data.

## Data Availability Statement

The 16S rRNA gene amplicon sequencing data generated during the current study were submitted to NCBI under BioProject PRJNA750583.

## Ethics Statement

All animal experiments were performed according to the ethical policies and procedures approved by the Animal Care and Use Committee of Yangzhou University, Jiangsu, China (Approval no. SYXK (Su) 2017-0044).

## Author Contributions

MW, LH, and YZ designed research; QX, LH, WW, JH, and YZ conducted research; YZ and LG, analysed data; YZ and LG wrote paper; MW, JJL, QY, and PZ reviewed and modified paper. MW and PZ had primary responsibility for final content. All authors read and approved the final manuscript.

## Funding

This study was supported by grants from the Natural Science Foundation of China (31672446); the funds from State Key Laboratory of Sheep Genetic Improvement and Healthy Production (2021ZD07; 2021ZD01; SKLSGIHP2021A03), Shihezi, China; and the Priority Academic Program Development of Jiangsu Higher Education Institutions, China.

## Conflict of Interest

The authors declare that the research was conducted in the absence of any commercial or financial relationships that could be construed as a potential conflict of interest.

## Publisher’s Note

All claims expressed in this article are solely those of the authors and do not necessarily represent those of their affiliated organizations, or those of the publisher, the editors and the reviewers. Any product that may be evaluated in this article, or claim that may be made by its manufacturer, is not guaranteed or endorsed by the publisher.

## Supplementary Material

Supplementary Table 1 Specific primers used for RT-PCR.

Supplementary Figure 1 Representative western blots of Per2 and Gapdh protein in KO and CON mice under NLD and SL.

Supplementary Figure 2 The prediction of the KEGG primary pathway *via* PICRUSt2 in KO and CON mice under NLD and SL.
