# Supplementary material for: Normal Light-Dark and Short-Light Cycles Regulate Intestinal Inflammation, Circulating Short-chain Fatty Acids and Gut Microbiota in Period2 Gene Knockout Mice
Source: Front Immunol. 2022 Mar 18;13:848248. doi: 10.3389/fimmu.2022.848248 (PMC8971677; doi:10.3389/fimmu.2022.848248)

**Normal Light-dark and Short-Light Cycles Regulate Intestinal Inflammation, Circulating Short-chain Fatty Acids and Gut Microbiota in *Period2* Gene Knockout Mice**

**Supplementary Materials**

**Table S1: Specific primers used for RT-PCR**

| Name | Primer Name | Sequence (5’-3’) | GenBank accession |
| --- | --- | --- | --- |
| *Per2* | *Per2*-F | TGTGCGATGATGATTCGTGA | XM_006529250.5 |
|  | *Per2*-R | GGTGAAGGTACGTTTGGTTTGC |  |
| *Per1* | *Per1*-F | GAAACGGCAAGCGGATGGA | NM_011065.5 |
|  | *Per1*-R | GGAAGGTGAAGAAGCCACCA |  |
| *Per3* | *Per3*-F | AAAAGATCCTGACCTCGCCC | NM_001289877.1 |
|  | *Per3-*R | AGTGGACCCTGCTTGAACAC |  |
| *Cry1* | *Cry1*-F | CACTGGTTCCGAAAGGGACTC | NM_007771.3 |
|  | *Cry1*-R | CTGAAGCAAAAATCGCCACCT |  |
| *Cry2* | *Cry2*-F | GGTTCCGCAAAGGACTACGG | NM_009963.4 |
|  | *Cry2*-R | ATCGGTTGATGCCCACAGAC |  |
| *Clock* | *Clock*-F | CTTCCTGGTAACGCGAGAAAG | XM_011249402.3 |
|  | *Clock*-R | TCGAATCTCACTAGCATCTGACT |  |
| *Bmal1* | *Bmal1*-F | CCACCTCAGAGCCATTGATACA | XM_030242022.2 |
|  | *Bmal1*-R | GAGCAGGTTTAGTTCCACTTTGTCT |  |
| *Rev-erbα* | *Rev-erbα*-F | TACATTGGCTCTAGTGGCTCC | NM_145434.4 |
|  | *Rev-erbα*-R | CAGTAGGTGATGGTGGGAAGTA |  |
| *Nhe1* | *Nhe1*-F | GAAGATAGGCAAGGAACCCGA | NM_001358455.1 |
|  | *Nhe1*-R | GAGATGGTGGGGATCACATGG |  |
| *Nhe2* | *Nhe2*-F | CAGGGCTTCCACTTCAACCT | NM_001033289.2 |
|  | *Nhe2*-R | TCCGAGTCGCTGCTATTTCC |  |
| *Nhe3* | *Nhe3*-F | GTGACTGGCGTGGATTGT | NM_001081060.2 |
|  | *Nhe3*-R | AGCAGGAAGGCGAAGATA |  |
| *Mct1* | *Mct1*-F | AGTGCAACGACCAGTGAAGT | NM_009196.4 |
|  | *Mct1*-R | GCGATCATTACTGGACGGCT |  |
| *Mct4* | *Mct4*-F | CAAGGTGCCTGAGTCTTCCTAA | NM_030696.3 |
|  | *Mct4*-R | ATGGTGTGCTGCCAAACAGTA |  |
| *Ae2* | *Ae2*-F | AAACATCTCAGCGGGCTCTC | NM_009207.3 |
|  | *Ae2*-R | CTATCCACCTCCAGTCGGGT |  |
| *NA^+^/K^+^ ATPase* | *NA^+^/K^+^ ATPase* -F | AGCCGAGGATTAACACCTGC | NM_144900.2 |
|  | *NA^+^/K^+^ ATPase* -R | TCTGTAGCACTTCGGATGCC |  |
| *Il-1β* | *Il-1β*-F | TGCCACCTTTTGACAGTGATG | NM_008361.4 |
|  | *Il-1β*-R | ATGTGCTGCTGCGAGATTTG |  |
| *Il-6* | *Il-6-*F | TGATGGATGCTACCAAACTGGA | NM_001314054.1 |
|  | *Il-6*-R | GTGACTCCAGCTTATCTCTTGGT |  |
| *Tnf-α* | *Tnf-α-*F | ACTGAACTTCGGGGTGATCG | NM_001278601.1 |
|  | *Tnf-α*-R | TGGTGGTTTGTGAGTGTGAGG |  |
| *Zo-1* | *Zo-1-*F | GCCGCTAAGAGCACAGCAA | XM_036152895.1 |
|  | *Zo-1*-R | TCCCCACTCTGAAAATGAGGA |  |
| *Claudin-1* | *Claudin-1-*F | GGGGACAACATCGTGACCG | NM_016674.4 |
|  | *Claudin-1*-R | AGGAGTCGAAGACTTTGCACT |  |
| *Occludin* | *Occludin-*F | TGAAAGTCCACCTCCTTACAGA | NM_001360538.1 |
|  | *Occludin*-R | CCGGATAAAAAGAGTACGCTGG |  |
| *Tlr2* | *Tlr2*-F | ACCCGCCCTTTAAGCTGTGT | NM_011905.3 |
|  | *Tlr2*-R | TCGTACTTGCACCACTCGCT |  |
| *Tlr4* | *Tlr4-*F | TCTGGGGAGGCACATCTTCT | NM_021297.3 |
|  | *Tlr4*-R | AGGTCCAAGTTGCCGTTTCT |  |
| *Myd88* | *Myd88*-F | ACTGGCCTGAGCAACTAGGA | NM_010851.3 |
|  | *Myd88-*R | CGTGCCACTACCTGTAGCAA |  |
| *Nf-κb p65* | *Nf-κb p65*-F | GAGGCACGAGGCTCCTTTTCT | XM_006531695.3 |
|  | *Nf-κb p65*-R | GTAGCTGCATGGAGACTCGAACA |  |
| *Gaphd* | *Gaphd*-F | AGGTCGGTGTGAACGGATTTG | NM_001289726.1 |
|  | *Gaphd* -R | TGTAGACCATGTAGTTGAGGTCA |  |

Figure S1: Representative western blots of Per2 and Gapdh protein in KO and CON mice under NLD and SL.


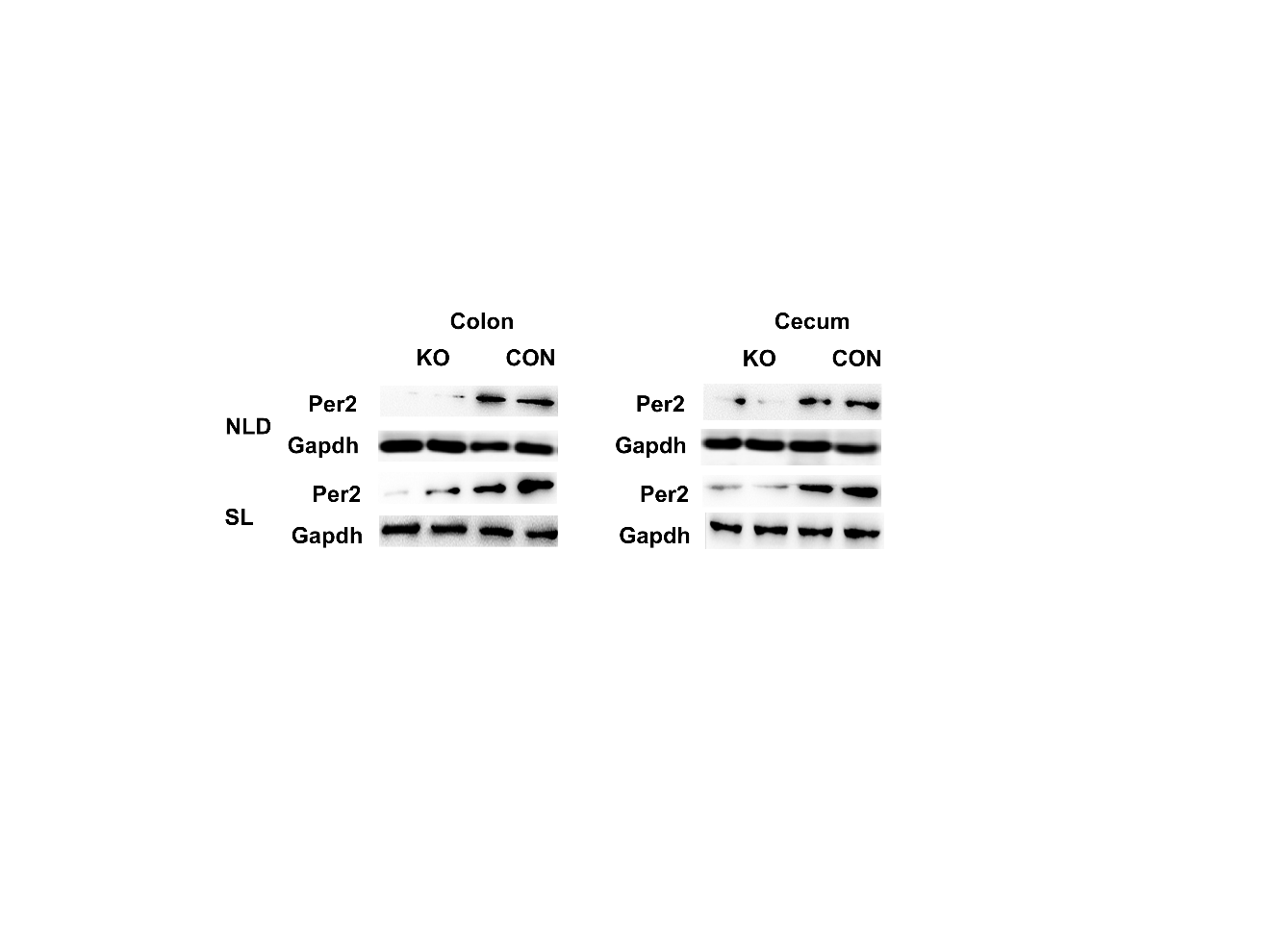


Figure S2: The prediction of the KEGG primary pathway *via* PICRUSt2 in KO and CON mice under NLD and SL.


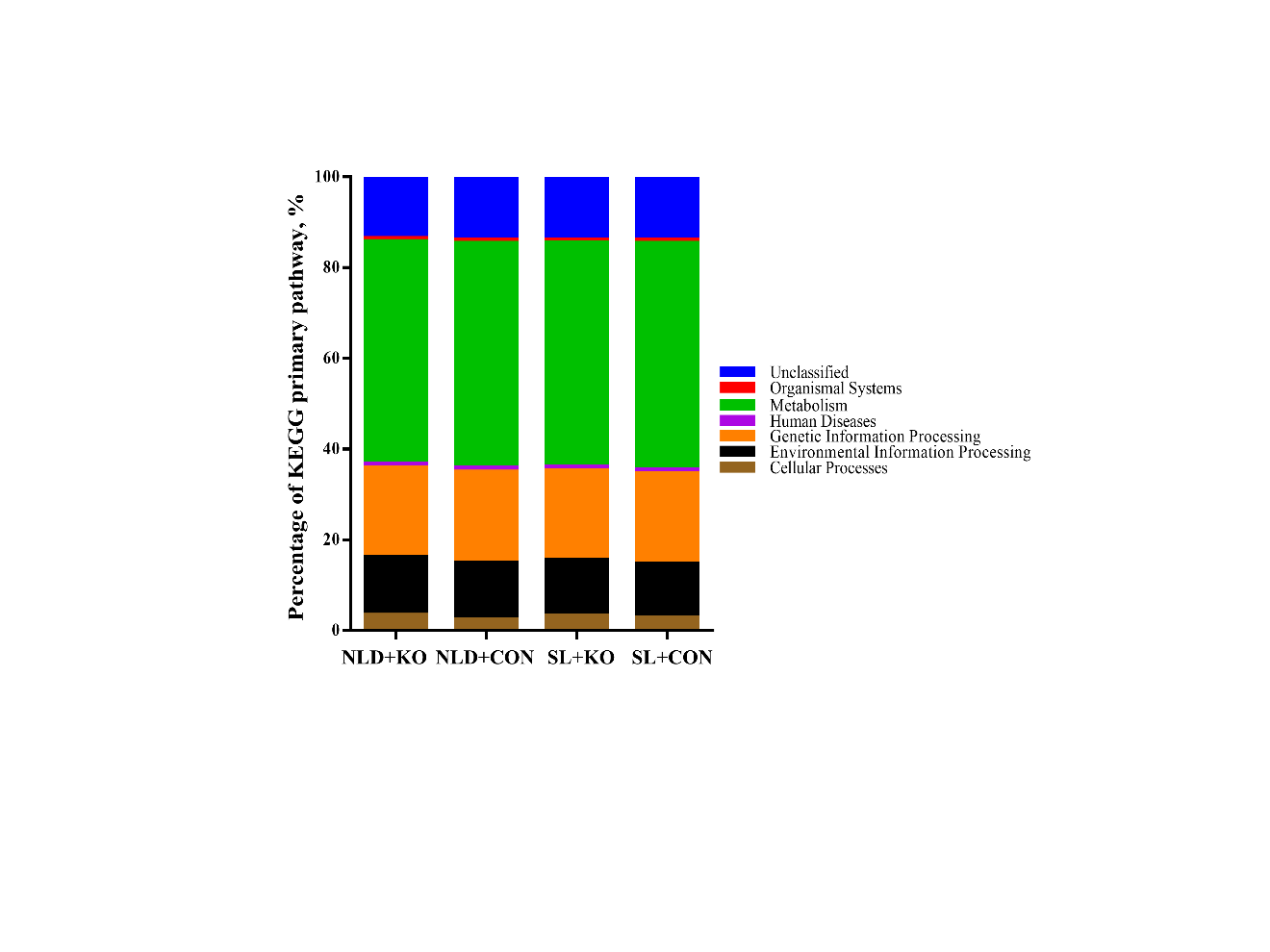

Supplement: Supplementary Table 1 — Specific primers used for RT-PCR. [file DataSheet_1.docx]
